# Unravelling the Complexity of Congenital Tracheal Stenosis with ‘O’ Rings

**DOI:** 10.1093/ejcts/ezaf478

**Published:** 2025-12-19

**Authors:** Athena Eliana Arsie, Marie Lamassiaude, Riccardo Nocini, Kishore Sandu

**Affiliations:** Department of Otolaryngology-Head and Neck Surgery, University of Verona, Verona 37134, Italy; Department of Otorhinolaryngology, University Hospital—CHUV, Lausanne 1005, Switzerland; Department of Otolaryngology-Head and Neck Surgery, University of Verona, Verona 37134, Italy; Department of Otorhinolaryngology, University Hospital—CHUV, Lausanne 1005, Switzerland

**Keywords:** circular rings, trachea, slide tracheoplasty

## Abstract

**Objectives:**

Congenital tracheal stenosis (CTS) in paediatric patients is a rare condition, accounting for only 0.3% to 1% of all laryngotracheal stenoses. It is characterised by complete cartilaginous ‘O’-shaped tracheal rings replacing the normal posterior membranous portion. This monocentric case series analyses management strategies and assesses surgical and functional outcomes.

**Methods:**

Twenty-one patients (12 males, 9 females) underwent surgery for CTS. Nineteen patients received slide tracheoplasty, while two underwent tracheal resection with primary anastomosis. Pre- and postoperative assessments focused on respiratory, swallowing, and vocal functions. The study also examined correlations between prior non-airway surgeries, extent of stenosis, and postoperative complications.

**Results:**

All patients demonstrated postoperative improvement in respiratory, swallowing, and vocal functions. Wilcoxon signed-rank testing revealed a statistically significant improvement in respiratory scores, whereas improvements in voice scores did not reach statistical significance. Swallowing scores showed no variation pre- and postoperatively and were thus excluded from this analysis. A significant positive correlation was observed between cardiopulmonary bypass duration and postoperative intubation time (Pearson’s r = 0.585, 95% CI 0.205-0.812, *P* = .007). In contrast, correlations between ICU length of stay and complication severity (Spearman’s ρ  =  0.316, 95% CI −0.113 to 0.787, *P* = .175) as well as between patient age at surgery and complication severity were not statistically significant. Additionally, linear associations were identified between prior non-airway surgeries and increased complication rates and between bypass time, hospital stay duration, and complication severity.

**Conclusions:**

Slide tracheoplasty remains the preferred technique for long-segment CTS, offering structural and functional restoration. Short-segment disease can be effectively managed with segmental resection and anastomosis. Prognosis could be influenced by stenosis length, associated comorbidities, and prior interventions, but larger studies would be needed to achieve adequate statistical power. Careful preoperative evaluation, tailored surgical planning, and attentive postoperative care are vital for optimal outcomes. Surgical intervention may be warranted even in select mildly symptomatic patients to prevent progression to life-threatening airway compromise.

## Graphical abstract

**Figure:**
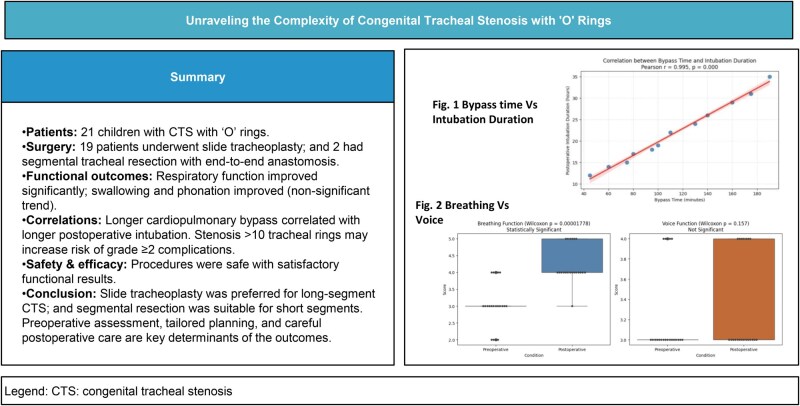

## Introduction

**Figure 1.**
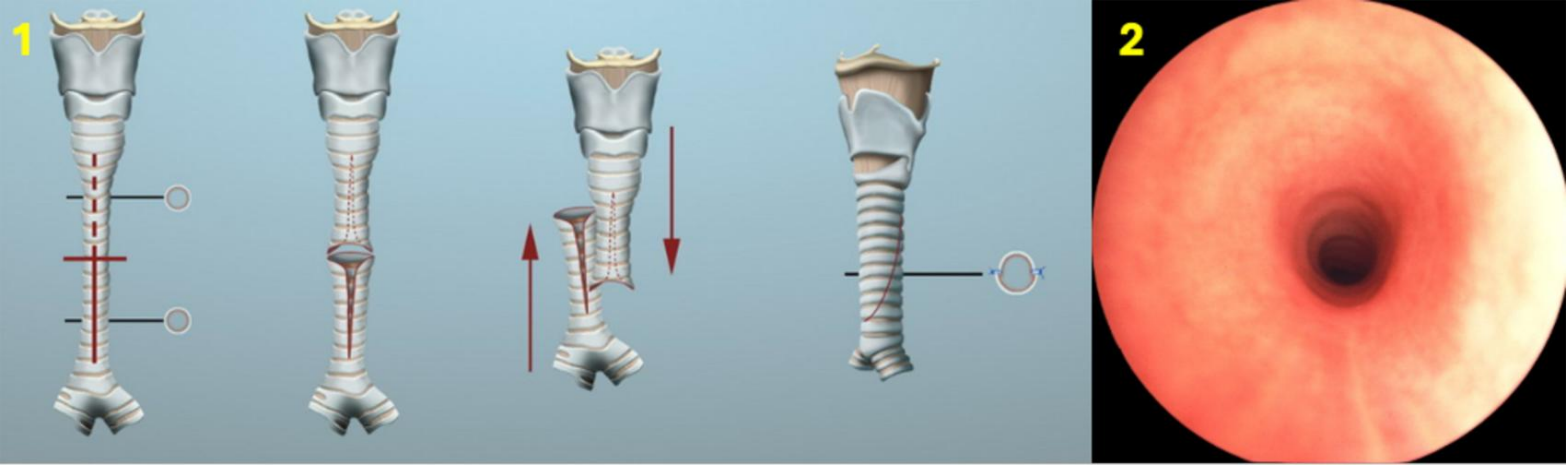
Schema of slide tracheoplasty. (1) rigid endoscopy view of ‘O’ ring trachea (2).

**Figure 2.**
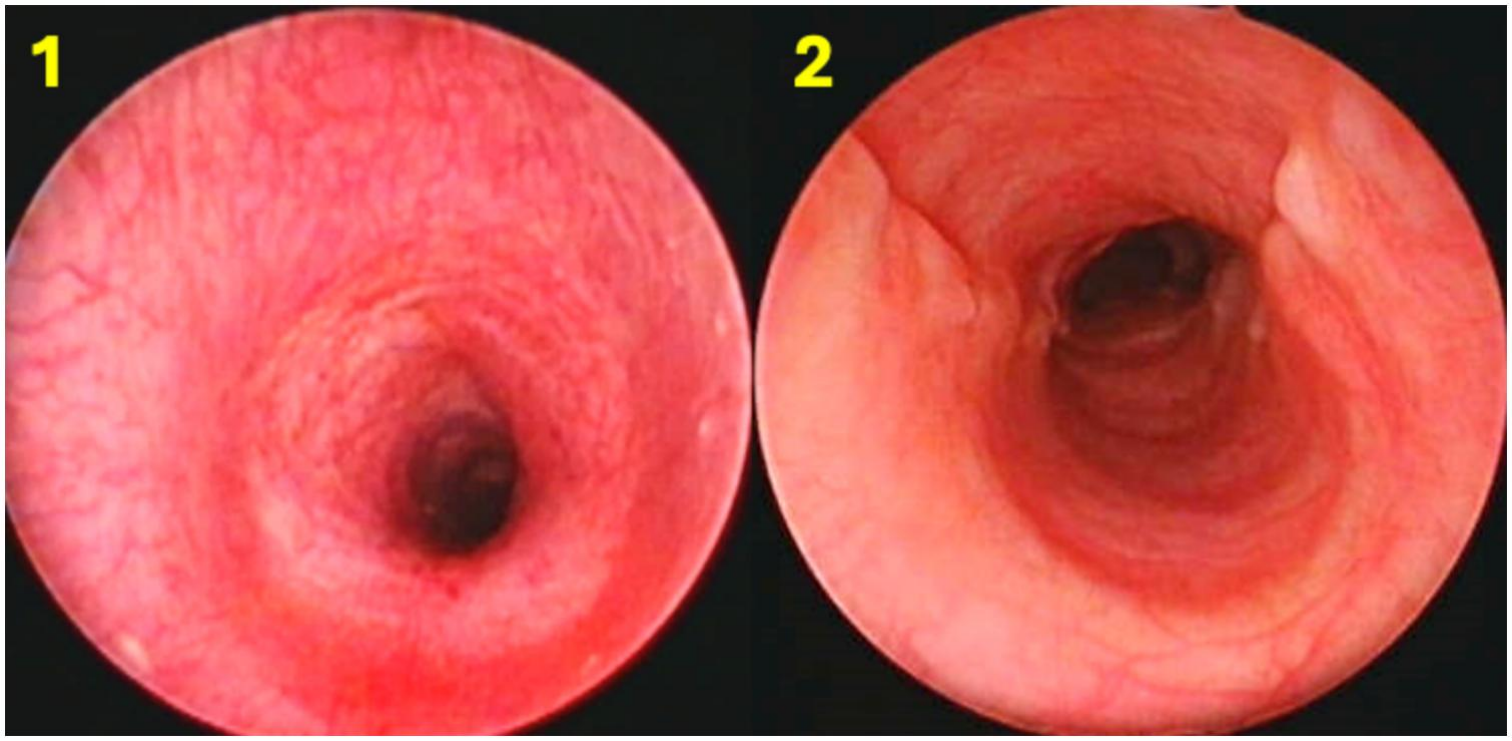
Endoscopic views pre- and post-slide tracheoplasty. Congenital ‘O’ ring trachea with complete ‘O’ rings (1) and following a slide tracheoplasty (2).

Paediatric tracheal stenosis can be classified as congenital or acquired, further divided by segment length into long or short stenosis. Congenital tracheal stenosis (CTS) occurs in about 1 in 64 500 cases^1^ and represents only 0.3% to 1% of all laryngotracheal stenoses. Complete tracheal rings, resulting from an embryological defect after the eighth week of gestation, form a fully cartilaginous ‘O’-shaped ring replacing the normal posterior membranous trachea.^2^^,^^3^ This anomaly causes tracheal narrowing with variable symptoms; most infants present severe respiratory distress, including biphasic ‘washing machine’ stridor, retractions, increased work of breathing, and cyanotic spells. Common comorbidities include pulmonary artery sling, tracheal bronchus, Down syndrome, and VACTERL variants.^3^ Diagnosis is sometimes incidental or mistaken for asthma in young adults. Since tracheal growth often lags behind somatic growth, and therefore symptoms typically appear as airway development falls behind.^4^^,^^5^ Surgical options include segmental resection with re-anastomosis,^6^^,^^7^ augmentation tracheoplasty using costal cartilage,^8^ pericardial patches,^9^ tracheal autografts,^10^ allografts,^11^ and laser division with balloon dilation.^12^ Slide tracheoplasty (ST) (**Figure 1**) involves dividing the stenotic segment at its midpoint, making anterior and posterior vertical incisions on opposing segments, trimming corners, then sliding and suturing them together. This doubles the tracheal circumference and quadruples the cross-sectional area while halving the stenotic length, restoring native cartilage and epithelium (**Figure 2**). Mortality rates reported by Butler et al^13^ and Manning et al^14^ are 6% and 2.5%, respectively, in large series of ST patients. Since Tsang et al^15^ introduction and Grillo’s popularization, ST is the preferred repair for complete rings due to its native tissue reconstruction, potential for growth,^16^^,^^17^ and versatility in complex cases like tracheal bronchus or lung hypoplasia. This report presents a single-centre case series highlighting the surgical decision-making process, functional outcomes, and key management considerations for CTS patients.

## Methods

Between 2014 and 2025, 21 patients underwent major surgery for Congenital Tracheal Stenosis (CTS) caused by complete ‘O’ rings at a tertiary referral university hospital specializing in paediatric airway management. All surgeries were performed by the senior author (KS). Data collected included demographics (gender, prior treatments, age at diagnosis and surgery), stenosis details (number of ‘O’ rings), preoperative status (comorbidities, ventilation history, vocal fold mobility), postoperative outcomes (complications, management, ICU stay), and functional assessments (breathing, feeding, voice) before and after surgery. Most patients (*n* = 14) were referred from international centres, with 7 from other national university hospitals. Patients were clinically stratified using a custom scoring system based on respiration, swallowing, and phonation. Respiratory status ranged from 1 (normal) to 4 (limited at rest); phonation from 1 (normal voice) to 4 (severe dysphonia); swallowing from 1 (normal) to 3 (moderate to severe dysphagia requiring feeding support). Scores from 1 to 3 or 4 were assigned for each variable, with higher values indicating worse function. This customized system was chosen due to the retrospective nature and external referrals, rather than standardized questionnaires. In our practice, the choice between cardiopulmonary bypass (CPB) and extracorporeal membrane oxygenation (ECMO) is based on the presence or absence of associated cardiac anomalies. CPB is used when concomitant intracardiac repair is required, as it allows correction of the cardiac defect and provides cardiac decompression, thereby improving exposure of the trachea during reconstruction. In contrast, venoarterial ECMO (VA-ECMO) is preferred in patients without cardiac anomalies, as it offers adequate cardiopulmonary support and stable gas exchange while avoiding the full systemic effects, anticoagulation requirements, and inflammatory response associated with CPB.

We used the Clavien Dindo classification^18^ of complications, graded from 0 (none) to 5 (death), with increasing severity (mild respiratory distress to cardiac arrest). All patients had at least one predischarge endoscopy at the centre. Postoperative evaluations by speech and swallow therapists were communicated to referring centres. Follow-up data were collected through email exchanges and online meetings with physicians and families and maintained in an ongoing registry. We defined the Age Appropriate Airway (AAA) as an airway diameter corresponding to the expected normal tracheal size for the patient’s chronological age, based on established paediatric airway growth charts or reference values^19^^,^^20^ At the last endoscopy, an optimal AAA was defined as an airway diameter ≥ 70% of the expected age-appropriate diameter with substantial clinical improvement, while suboptimal AAA was defined as an airway diameter < 70% of the expected age-appropriate diameter. This report includes data throughout the study period up to the latest follow-up by the treating physicians. This study is a retrospective, descriptive analysis with a limited sample size, and no data were missing. The small sample size is consistent with the rarity of the condition, making it impossible to apply advanced statistical models.

## Results

### Characteristics of the study population (Tables 1 and 2)

The study included 21 patients (9 females, 12 males) aged 3-108 months (median = 25 months, IQR 1.5-61), diagnosed at a median age of 29.5 months (range 1-95). Seventeen had cardiopulmonary anomalies, including Tetralogy of Fallot (*n* = 5), vascular anomalies like pulmonary artery sling or aberrant subclavian artery, and minor cardiac defects (VSD, ASD; *n* = 7). Four patients exhibited laryngomalacia or tracheobronchomalacia, with one also having obstructive sleep apnea in VACTERL syndrome. Preoperative bronchoscopy revealed 3–17 complete tracheal rings. Nineteen patients underwent slide tracheoplasty (ST) via cervico-sternotomy; two had tracheal resection-anastomosis (TRA) due to fewer than four rings. One required bronchoplasty extending the slide into the narrowed right main bronchus, and two with Grade II subglottic stenosis had the slide extended into the cricoid. Most stenoses were congenital (*n* = 18), two were mixed (associated with intubation). Mean surgery age was 36.6 months (SD 30,6). Half (*n* = 11, 52%) had prior non-airway surgery and were diagnosed with CTS during endoscopy for persistent symptoms—ten cardiac, one abdominal. An 8-year-old girl with untreated CTS presented with a critical airway during an RSV infection. Six patients required preoperative intubation, all had respiratory grades 2-3 at diagnosis.

**Table 1.** Patients’ Characteristics

Patient’s ID	Sex	Age at diagnosis (month)	National/International (N/I)	Previous non-airway surgery	Other procedures during tracheal surgery	Synchronous cardiac-vascular anomaly
1	M	86	I	No	Yes	TOF
2	F	42	N	Yes	No	Common arterial trunk
3	M	0	I	No	No	TOF
4	F	84	N	Yes	No	TBM
5	F	0	I	No	No	VSD
6	M	13	I	No	No	VSD, ASD
7	F	50	I	No	No	None
8	F	31	I	No	Yes	TOF
9	M	25	I	No	Yes	TOF
10	M	20	I	No	Yes	TBM
11	M	5	N	Yes	Yes	Left pulmonary artery sling
12	M	0	N	Yes	NA	None
13	M	12	N	Yes	Yes	OSAS in VACTERL syndrome
14	M	0	I	Yes	NA	Left pulmonary artery sling
15	F	2	I	Yes	No	VSD, ASD, Aberrant subclavian artery
16	F	95	I	Yes	No	TOF
17	F	1	I	Yes	Yes	TBM
18	M	25	N	No	No	TBM
19	M	32	N	Yes	No	VSD, ASD, Aberrant subclavian artery
20	F	72	I	No	No	None
21	M	108	I	Yes	NA	VSD

ASD, Atrial Septal Defect; OSAS, obstructive sleep apnea syndrome; TBM, Tracheobroncomalacia; TOF, Tetralogy of Fallot; VACTERL syndrome, Vertebral defects, Anal atresia, Cardiac defects, Tracheo-Oesophageal fistula, Renal anomalies, and Limb abnormalities; VSD, Ventricular Septal Defect.

**Table 2.** Stenosis Characteristics and Surgical Details

Patient ID	Stenosis characteristics		Surgical details					
	Congenital/Acquired/Mixed (C/A/M)	Number of rings involved	Age at airway surgery (months	Surgery technique	CPB time (mins)	ICU stay (days)	Complications rate	Final outcome Age-Appropriate Airway (AAA) O: optimal; SO: suboptimal
1	C	9	84	ST	135	18	1	O
2	C	8	24	ST	105	5	1	O
3	M	6	36	ST + anterior cricoid expansion	100	11	0	O
4	C	14	84	ST	180	7	0	O
5	C	6	94	ST	320	8	0	SO
6	C	16	16	ST + anterior cricoid expansion	425	21	2	SO
7	C	6	48	ST	125	4	0	O
8	C	14	36	ST	200	16	2	SO
9	C	16	24	ST + bronchoplasty	364	26	1	O
10	C	15	20	ST	267	11	2	SO
11	M		5	ST	363	49	3	SO
12	C	9	5	ST	170	9	0	O
13	C	3	16	TRA	165	14	1	O
14	C	11	9	ST	100	15	0	O
15	C	14	3	ST	170	7	0	O
16	C	9	96	ST	180	6	2	O
17	C	17	24	TRA	181	5	3	SO
18	C	11	25	ST	130	9	0	O
19	C	12	20	ST	187	85	4*	SO
20	C	14	72	ST	162	8	0	O
21	C	16	52	ST	105	9	0	O

Degree of complications: (0) none, (1) Mild respiratory distress, (2) Major respiratory distress, (3) ECMO or shock, (4) Cardiac arrest (CA), (5) Death. *#19 needed a temporary tracheostomy for partial tracheal necrosis.

CPB, cardiopulmonary bypass time; ST, Slide Tracheoplasty; TRA, Tracheal Resection and anastomosis.

### ICU admission and postoperative complications (Table 2)

**Figure 3.**
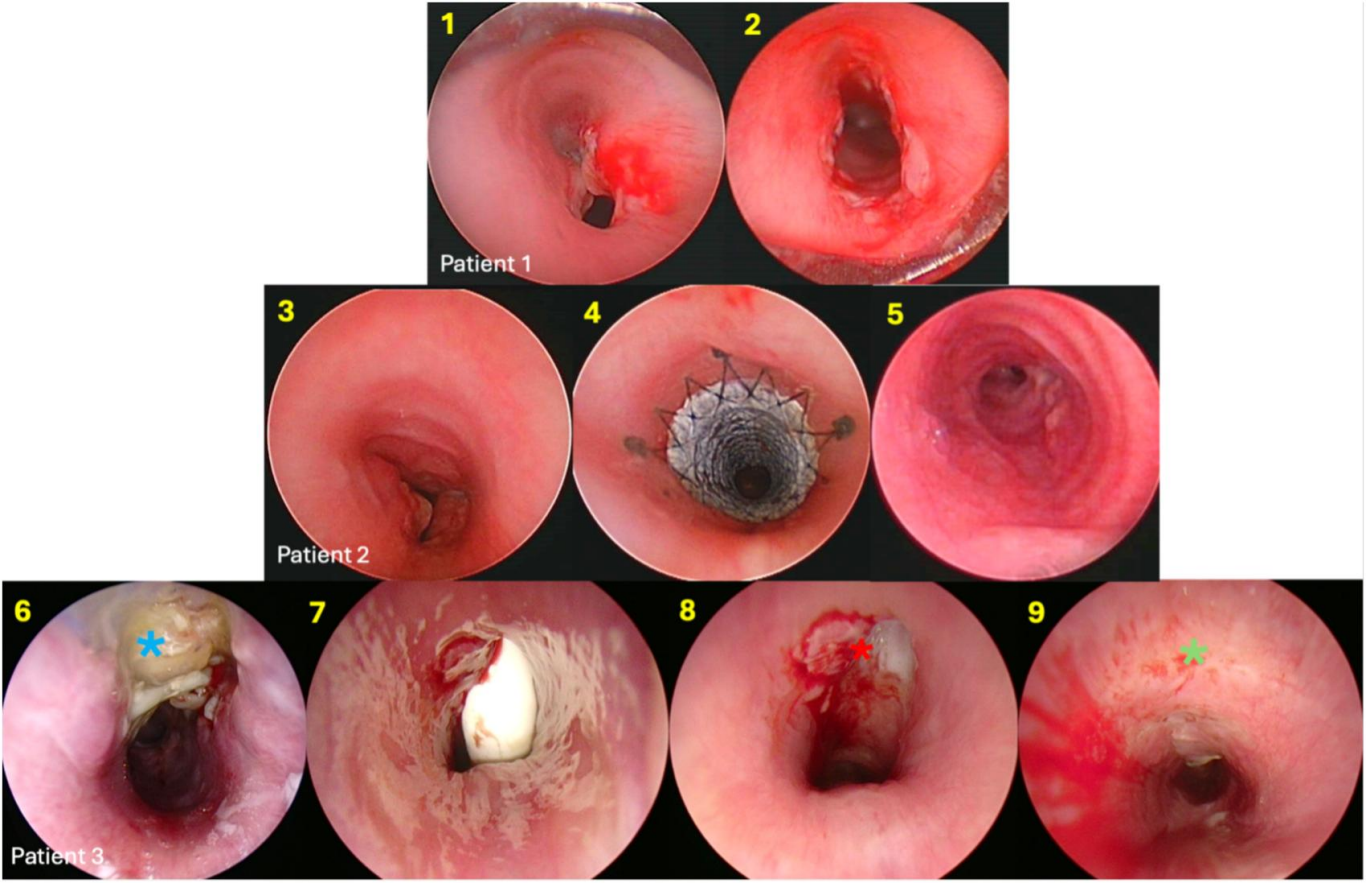
Complications following slide tracheoplasty. Patient 1: An 8-shape deformity (1) following a slide tracheoplasty. Correction of the complication was done using CO2 laser and balloon dilation (2). Patient 2: Significant tracheal stenosis (3) was treated with temporary placement of Fluency Plus vascular stent (4) (Angiomed Gmbh & Co. Medizintechnik, Karlsruhe, Germany) with a favourable result (5). Patient 3: The child (patient #19) had a very complex single ventricle and lung condition. (6) Complete necrosis of the anterior half of the trachea (blue asterisk corresponds to the defect. In our practice, intraoperatively we routinely use the thymus between the trachea and the innominate vessels). The child needed a tracheostomy (7, 8 red asterisk). The anterior defect eventually healed satisfactorily around the cannula (9, green asterisk) and the tracheal dimensions were favourable. The child was eventually decannulated.

Postoperative data showed a mean ICU stay of 17.2 days (range 4-85, SD 18,6) and mean intubation duration of 6.55 days (range 1-17, SD 4,8). Median follow-up was 6.2 months (0.5-36), with a mean of four postoperative endoscopies per patient (range 2-17, SD 3,3). Minor-major complications occurred in 11/21 patients (4 patients/19% were airway-related, 7/33% were non-airway related). Two patients with cardiac comorbidities needed resuscitation, and we report no deaths. Four patients had minor granulations needing only ablation. Restenosis in form of an ‘8’-shaped deformity occured in seven patients needing laser ablation and endoscopic balloon dilation (**Figure 3**, patient 1). Three patients required temporary stents (**Figure 3**, patient 2), among which one had a partial anastomotic dehiscence. Another patient had partial tracheal necrosis needing temporary tracheostomy (**Figure 3**, patient 3). An exploratory logistic regression was conducted to examine whether stenosis length, CPB time, or presence of previous non-airway surgery were associated with clinically relevant complications (≥ grade 2 Clavien- Dindo classification). Given the small sample size, these analyses are considered exploratory, and descriptive data are provided to summarize the data without overinterpreting potential associations.

### Extent of tracheal stenosis

Our data indicates a trend suggesting that the length of the stenotic segment (affected tracheal rings > 10) may influence the postoperative prognosis, specifically for complications of degree 2 and higher. However, this result was not statistically significant due to the large standard deviation in the stenosis characteristics. It is important to note that, due to the limited sample size, we are unable to establish causality between these factors but can only identify potential correlations. A Pearson correlation analysis revealed a strong, though not perfect, positive linear correlation between some variables. We found a strong correlation between postoperative intubation duration and cardiopulmonary bypass time, suggesting that longer bypass durations are associated with longer intubation periods (Pearson’s r = 0.585, 95% CI 0.205-0.812, *P* = .007). Additionally, we calculated the Spearman correlation between ICU stay and the degree of postoperative complications, though it was not statistically significant (Spearman’s ρ  =  0.316, 95% CI −0.113 to 0.787, *P* = .175). Similarly, we did not find any correlation between the age at the time of surgery and the severity of complications.

### Surgery outcomes (Table 3)

In this cohort, 11 of 21 (52%) patients had prior non-airway surgeries (mainly cardiac) before tracheal correction for complete ‘O’ ring stenosis, associated with longer ICU stays and more severe postoperative complications. Point-Biserial correlation showed a weak positive link between prior surgery and ICU stay length, and a stronger correlation with complication severity (Point-Biserial r_pb = 0.205, 95% CI −0.249 to 0.585, *P* = .373). The most severe complications (grade 3-4, *n* = 3) occurred in patients with previous cardiac surgery; and two required additional cardiac procedures during tracheal surgery. No significant correlation was found between prior surgery and intubation duration, though it was longer (median: 7 vs. 2 days) in patients with previous surgical history. Restenosis affected 33% (7/21), mainly patients with major cardiopulmonary comorbidities like common arterial trunk and Tetralogy of Fallot; four needed temporary stents for ‘8’-shaped deformities. No patient required a revision open surgery. Majority patients (16/21, 76%) had an optimal to suboptimal age-appropriate airway at the last endoscopy.

**Table 3.** Surgical Outcomes

	Airway status						
Patient ID	Vocal Fold mobility	Number of endoscopies	Restenosis	Dehiscence	Granulation	Revision surgery Open(O)/Endoscopic (E)/Tracheostomy (T)	Type of revision surgery
1	0	6	Yes	No	1	Yes, E	Dilation + temporary stent
2	1	6	Yes	No	1	Yes, E	Dilation + temporary stent
3	1	2	No	No	1	No	/
4	2	2	No	No	1	No	/
5	1	2	No	No	1	Yes, E	Laser Ablation
6	0	4	No	No	1	Yes, E	Temporary stent
7	1	2	No	No	0	No	/
8	1	3	Yes	Yes	2	Yes, E	Dilation + temporary stent
9	0	2	No	No	2	Yes, E	Supraglottoplasty
10	0	3	No	No	0	No	/
11	2	7	Yes	No	2	Yes, E	Dilation (3 procedures)
12	1	6	Yes	No	0	Yes, E	Dilation (1 procedure)
13	1	4	Yes	No	0	Yes, E	Dilation (2 procedures)
14	0	3	No	No	1	No	/
15	0	5	No	No	0	No	/
16	2	2	No	No	0	No	/
17	1	2	No	No	1	No	/
18	1	4	No	No	1	No	/
19	0	17	Yes	No	0	T	/
20	1	3	No	No	0	No	/
21	1	2	No	No	0	No	/

Vocal Fold mobility: (0) information not available, (1) normal, (2) persistent unilateral VC palsy, (3) bilateral palsy.

Granulation: 0- no granulations, 1-minor granulations needing limited endoscopies(<2), 2-severe granulations needing > 2 endoscopies.

### Functional outcomes and follow-up

Many patients were referred from foreign countries, making clinical follow-up challenging. We obtained feedback from colleagues managing the patients in their countries of origin, who then reported any emerging issues to us. **Table 4** provides updated data on the patients’ functional status. In most of them (19 out of 21; 90%), the postoperative functional score was better than the preoperative score, indicating clinical improvement. Seven patients developed postoperative restenosis, which was managed successfully with endoscopic treatment, and all showed improved functional scores. One patient (Patient 9) had no change in score due to left arytenoid subluxation, which required laser resection. Another (Patient 17) had pre-existing left vocal cord paralysis from a previous surgery, but this did not negatively affect the postoperative outcome. Detailed information is provided in **Table 4**. We applied the Wilcoxon signed-rank test to the pre- and post-operative data for breathing, voice, and swallowing; the analysis revealed that there is a statistically significant difference in the improvement of breathing scores (*P* = .001), but not in voice scores (*P* = .083). For swallowing, the test could not be applied because there were no changes in values between paired data. At the latest follow-up, 14 patients (66%) had a tracheal diameter appropriate for their age. In 7 patients (33%), although the tracheal size was suboptimal and below age expectations, there were no activity limitations, and no further endoscopic interventions were required. One patient (Patient 19) developed partial tracheal necrosis requiring a tracheostomy but has since been decannulated. He is currently undergoing post cardiac arrest rehabilitation.

**Table 4.** Functional Outcomes

Patient’s ID	Score								
	Pre-operative			Post-operative			Pre-OP results	Post-OP results	Gain for the score
	Pre-OP breathing	Pre-OP voice	Pre-OP deglutition	Post-OP breathing	Post-OP voice	Post-OP deglutition			
1	2	2	1	2	1	1	5	4	Yes
2	3	1	1	2	1	1	5	4	Yes
3	3	2	1	1	1	1	6	3	Yes
4	3	1	1	1	1	1	5	3	Yes
5	3	1	1	1	1	1	5	4	Yes
6	2	1	1	1	1	1	4	3	Yes
7	3	1	1	1	1	1	5	3	Yes
8	3	1	1	1	1	1	5	3	Yes
9	3	1	1	2	1	1	5	3	Yes
10	3	2	1	1	1	1	6	3	Yes
11	3	1	1	1	1	1	5	3	Yes
12	2	1	1	1	1	1	4	3	Yes
13	2	1	1	2	1	1	4	5	No
14	2	1	1	1	1	1	4	3	Yes
15	2	1	1	1	1	1	4	2	Yes
16	2	1	1	1	1	1	4	3	Yes
17	3	1	1	1	1	1	5	2	Yes
18	2	1	1	1	1	1	4	3	Yes
19	3	1	1	1	1	1	5	5	No
20	3	1	1	1	1	1	5	3	Yes
21	3	1	1	1	1	1	5	2	Yes

Breathing: 1. normal, 2. limited with forced exertion, 3. limited with moderate exertion, 4. limited at rest; Voice - 1. normal voice, 2. mild dysphonia/hoarse voice, 3. moderate dysphonia/weak voice or ventricular band phonation with easy fatigability, 4. severe dysphonia; Swallowing/deglutition -1. normal, 2. mild dysphagia (oral feeding possible but difficult with some textures, and no aspiration), 3. moderate to severe dysphagia requiring feeding support.

## DISCUSSION

Managing CTS with complete tracheal rings requires a holistic and multidisciplinary approach. The key findings in our study were: (1) Half of the patients were found to have CTS missed during previous non-airway surgeries, mainly cardiac; and (2) these patients required longer cardiopulmonary bypass times, experienced greater technical difficulty during slide tracheoplasty, had prolonged ICU stays, and developed more complications.

A comprehensive aero-digestive endoscopy is essential as important laryngo-tracheo-bronchial information may be missed by radiological exams. Specifically, an airway endoscopy is performed under spontaneous ventilation and total intravenous anaesthesia using a target-controlled infusion of propofol and topical lidocaine,^21^ and it offers the following critical information: (1) the exact site and the number of complete rings, (2) identification of the narrowest point of the airway and its diameter, (3) the number and location of the remaining normal tracheal rings and bronchial anatomy, (4) associated congenital subglottic stenosis or distal extension of complete rings in one- or both bronchi, (5) the quality of the tracheal mucosa and (6) the presence of synchronous aero-digestive lesions (eg, prior intubation lesions, obstructive sleep apnea, laryngomalacia, tracheo-bronchomalacia, gastroesophageal reflux disease), which must be addressed before performing corrective tracheal surgery. Failing to diagnose an associated CTS prior to a cardiac surgery is a significant error in the overall management, increasing the risk of intubation-related injuries and extubation failure, as was seen in three of our patients. During preoperative endoscopy, it is crucial to use slim telescopes that can pass through the stenotic portion of the trachea without causing iatrogenic mucosal trauma, which could potentially lead to the development of an acute airway obstruction in an already compromised airway. Intraoperatively, during slide tracheoplasty (ST), an appropriately sized flexible endoscope is passed through the endotracheal tube to identify the midpoint of the stenotic segment just before dividing the trachea.

Teamwork in airway management is fundamental, requiring a dedicated anaesthesiologist and clear, trusted collaboration with the surgeon through well-defined roles to ensure safe and effective patient care. Endoscopy serves both diagnostic and follow-up roles, using a 4 mm Storz endoscope to assess tracheal size and detect patients needing retreatment for restenosis, 8-shape deformity, or granulation tissue. The complications observed in our report were successfully treated only with endoscopic interventions and align with findings in the literature.^13^^,^^22^

What distinguishes our study from others is the focus on variables beyond just respiratory function during the follow-up period. In this context, we would like to highlight the excellent functional outcomes achieved by more than 2/3rd patients (76%) in this study.

Regarding the technique of ST, Tsang et al^17^ preferred dividing the proximal trachea posteriorly and the distal trachea anteriorly, while Rutter et al^5^^,^^14^ favoured the opposite approach, finding it more convenient, since the stenotic segment in CTS is conical and narrowest distally. In our practice, we usually prefer the Rutter technique, though, in an associated subglottic stenosis, we prefer the Tsang technique to facilitate easier anterior expansion of the cricoid cartilage. If the complete rings extend into both bronchi, we prefer anterior sliding of the proximal segment into only the narrowest bronchi. If a tracheostomy is necessary in patients diagnosed with complete ring trachea prior to trachea repair, we recommend a vertical incision in the proximal portion of the stenosis, to facilitate easy cannula insertion and using a small-size cannula to prevent trauma to the narrowest part of the trachea, which is typically located distally in most cases. To perform ST in presence of a prior tracheostomy, we prefer sliding the distal segment anteriorly to close the tracheostoma.

There is consensus in the literature that ST should be performed concurrently with the correction of associated cardiac malformations,^13^^,^^23–27^ offering the advantage of a single procedure and a single cycle of CPB, leading to improved post-operative cardiorespiratory stability. Yokoi et al^24^ reported that longer CPB times, extended tracheal stenosis segments, carina involvement, and prolonged mechanical ventilation have negative impacts on surgical outcomes, and this is in line with our findings. Prematurity, low birth weight, and complex cardiovascular malformations are poor prognostic factors,^23^ with pulmonary agenesis or hypoplasia also worsening outcomes due to higher mortality and postoperative complications. DeMarcantonio et al^25^ noted that having a single functional lung does not preclude surgical tracheal repair despite high mortality, and prolonged preoperative ventilation and ECMO predict worse postoperative results.^26^^,^^27^

While most children with severe tracheal stenosis need surgery, according to some authors^28^^,^^29^ about 17% patients with complete rings may be managed conservatively and suggested wait-and-watch policy when the narrowest tracheal diameter is ≥40% of normal. Our approach differs from the literature with respect to conservative management of CTS. We prefer surgery in otherwise healthy, minimally symptomatic patients who present with recurrent episodes of respiratory distress, despite responding well to conservative treatment, in order to prevent future life-threatening complications. This approach is supported by two cases: first was an adult patient with previously undiagnosed respiratory condition, and who required a tracheostomy due to COVID-19-related adult respiratory distress syndrome (ARDS). The inability to pass a tracheostomy cannula prompted an endoscopy, which confirmed the presence of long-segment CTS. Unfortunately, our inability to insert an adequately sized cannula, and therefore provide proper ventilation, led to her death. The second case (Case #20) was diagnosed with CTS at an external institution and the doctors preferred a conservative waiting. She developed a critical airway situation following an RSV infection while vacationing in our region, that required central ECMO placement. Following conservative treatment of the viral infection, we performed the ST. The patient recovered well and subsequently returned to her home country. We believe that this approach will help prevent future complications, enhance patients’ tolerance to infections, and support improved physical activity, including participation in sports and childbirth. Nonetheless, multicentre studies are needed to validate this approach, especially given the complexity of the surgery.

The trachea’s airway smooth muscles (ASM) enable dynamic respiratory function by modulating mechanical properties.^30^ Numerous studies have also shown that deep inspiration (DI) has both *bronchodilatory* and *bronchoprotective effects* on the ASM.^31^ Bronchodilation may result from actin-myosin cross-bridge disruption, while bronchoprotection involves smooth muscle plasticity, underscoring their role in respiratory dynamics. Based on respiratory mechanics and the absence of trachealis muscle in complete rings, we prefer tracheal resection and anastomosis (TRA) for short-segment CTS (<4 rings, 2 cases) to restore optimal ASM function, and especially because a short segmental resection should not increase complications like an anastomotic dehiscence. Clearly, a prospective multi-institutional study comparing TRA and ST for *short segments* is needed.

The main limitations of this study are the small sample size, which reflects the rarity of CTS, and the limited postoperative follow-up, as many patients were referred from international institutions and chose to continue their care at their parent hospitals.

## CONCLUSIONS

Management of CTS has evolved to emphasise a strategic, individualised approach, with factors like cardiopulmonary bypass duration affecting ICU stay and complications. Multidisciplinary team involvement is crucial for optimal outcomes. This study, despite including a limited number of CTS cases, presents novel insights that warrant further investigation.

## Data Availability

All data analysed during the present study are included in this article and have been extracted from electronic file of the patients.
